# Role of L-Type Voltage-Gated Calcium Channels in Epileptiform Activity of Neurons

**DOI:** 10.3390/ijms221910342

**Published:** 2021-09-25

**Authors:** Denis P. Laryushkin, Sergei A. Maiorov, Valery P. Zinchenko, Sergei G. Gaidin, Artem M. Kosenkov

**Affiliations:** 1 Institute of Theoretical and Experimental Biophysics of the Russian Academy of Sciences, 142290 Pushchino, Russia; mr.ldp@yandex.ru; 2 Federal Research Center “Pushchino Scientific Center for Biological Research of the Russian Academy of Sciences”, Institute of Cell Biophysics of the Russian Academy of Sciences, 142290 Pushchino, Russia; dikyagux@mail.ru (S.A.M.); vpz@mail.ru (V.P.Z.)

**Keywords:** voltage-gated calcium channels, nifedipine, isradipine, diltiazem, verapamil, neurons, paroxysmal depolarization shift, epileptiform activity

## Abstract

Epileptic discharges manifest in individual neurons as abnormal membrane potential fluctuations called paroxysmal depolarization shift (PDS). PDSs can combine into clusters that are accompanied by synchronous oscillations of the intracellular Ca^2+^ concentration ([Ca^2+^]_i_) in neurons. Here, we investigate the contribution of L-type voltage-gated calcium channels (VGCC) to epileptiform activity induced in cultured hippocampal neurons by GABA(A)R antagonist, bicuculline. Using KCl-induced depolarization, we determined the optimal effective doses of the blockers. Dihydropyridines (nifedipine and isradipine) at concentrations ≤ 10 μM demonstrate greater selectivity than the blockers from other groups (phenylalkylamines and benzothiazepines). However, high doses of dihydropyridines evoke an irreversible increase in [Ca^2+^]_i_ in neurons and astrocytes. In turn, verapamil and diltiazem selectively block L-type VGCC in the range of 1–10 μM, whereas high doses of these drugs block other types of VGCC. We show that L-type VGCC blockade decreases the half-width and amplitude of bicuculline-induced [Ca^2+^]_i_ oscillations. We also observe a decrease in the number of PDSs in a cluster and cluster duration. However, the pattern of individual PDSs and the frequency of the cluster occurrence change insignificantly. Thus, our results demonstrate that L-type VGCC contributes to maintaining the required [Ca^2+^]_i_ level during oscillations, which appears to determine the number of PDSs in the cluster.

## 1. Introduction 

Epilepsy is one of the most common neurological disorders worldwide. Approximately 1% of the global population (50–70 million people) have epilepsy [1]. According to the hypothesis of the excitation/inhibition balance in the brain, the general mechanism of epileptic seizures is a shift in the balance towards excitation [2]. These disturbances lead to the hyperactivation and hypersynchronization of neuronal ensembles. Epileptic discharges manifest in individual neurons as abnormal fluctuations in membrane potential called paroxysmal depolarization shift (PDS) [3,4]. Initially, these events were considered a neuronal correlate, describing interictal spikes registered with electroencephalography [4]. However, the current interpretation of PDS involves various epileptiform discharges, including epileptic bursts, segments of seizure-like activity, and post-ictal discharges, that occur in *in vitro* as well as *in vivo* models of epilepsy [5,6,7,8,9].

The described epileptiform activity can be induced by different types of exposure, shifting the E/I balance towards excitation, thus leading to network hyperexcitation. Picrotoxin [10], bicuculline [11], caffeine [3], and Mg^2+^-free medium [12] are commonly used inductors of epileptiform activity. The typical PDS is a positive depolarizing shift accompanied by single or several action potentials (APs). The amplitude of APs decreases alongside the decay of the depolarizing plateau. PDS occurs as a single electrical event with a 40–400 ms duration or as a cluster consisting of several rapidly repeated PDSs [4,13]. Individual PDS patterns can vary depending on the mechanism of induction, brain region, and neuron features [4,14,15].

Although PDS was first described in the 1960s, this phenomenon remains elusive [16,17]. Moreover, the role of PDS in epilepsy is disputable. There are hypotheses indicating pro- and antiepileptic effects of PDSs [18,19,20,21]. Additionally, this activity can be found in other neurological diseases [22,23,24]. Thus, a more detailed investigation of PDS generation and its spread mechanisms is essential in designing new therapeutic approaches and understanding the mechanisms underlying neurological disorders.

It is considered that Ca^2+^ inflow through L-type voltage-gated calcium channels (VGCC) is a critical step in the formation of PDS [25]. L-type VGCC Ca_v_1.1–Ca_v_1.4 are widely distributed in different mammalian cells. In particular, brain cells express predominantly Ca_v_1.2 and Ca_v_1.3 channels. These channels are also expressed in the heart tissue and adrenal glands [26]. The typical structure of an L-type calcium channel represents a heterotetramer consisting of a pore-forming transmembrane α1-subunit, intracellular β-subunits, and an extracellular α2δ-subunit. The α1-subunit determines the biophysical and pharmacological properties of the channel, and its topology is highly conservative [27]. Modulators of L-type VGCC can be roughly divided into two main groups: dihydropyridines and non-dihydropyridines. The group of non-dihydropyridines includes drugs from classes of phenylalkylamines, benzothiazepines, and diphenylpiperazines [28]. Although numerous blockers of L-type VGCC have been synthesized, the efficiency of these drugs was evaluated as a rule in cell lines where individual subunits were expressed. In this regard, only a few studies demonstrate the effects of these blockers under conditions close to physiological.

This study aimed to compare the effects of different L-type VGCC blockers and determine the contribution of L-type VGCC to the epileptiform activity of neurons. We used hippocampal neuroglial cell cultures in our studies since this cellular model allows the evaluation of the activity of numerous neurons united in one closed network. To induce epileptiform activity, we used bicuculline, an antagonist of GABA(A) receptors. Application of bicuculline suppresses GABA(A)R-mediated inhibition (disinhibition), thus promoting the hyperexcitation of a neuronal network. The dynamics of intracellular Ca^2+^ concentration ([Ca^2+^]_i_) and membrane potential were used as the main measured parameters to evaluate the effects of the studied drugs.

## 2. Results

### 2.1. Determination of Effective Concentrations of L-Type VGCC Blockers

**Figure 1:**
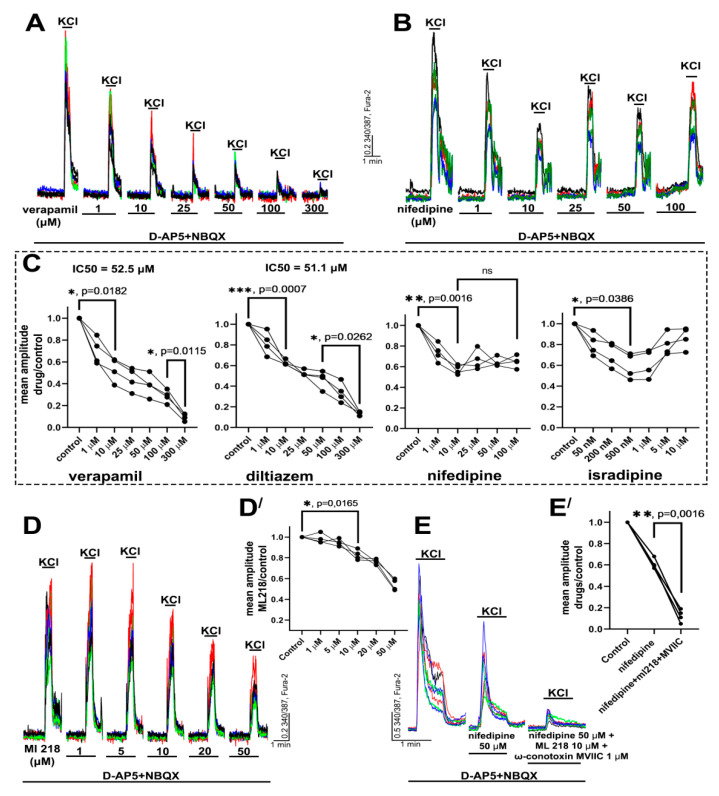
The effects of different VGCC blockers on KCl-induced Ca^2+^ response in neurons. (**A**,**B**,**D**) Effects of different doses of verapamil, nifedipine, and ML-218 (T-type VGCC blocker) on the amplitude of KCl-induced (35 mM) Ca^2+^ response in the presence of NMDAR (D-AP5, 10 μM) and AMPAR/KAR (NBQX, 10 μM) antagonists. The pauses between KCl applications were 15 min. The traces of some representative neurons are shown in each panel. N = 100, n = 4 for each experiment. (**C**,**D′**) Diagrams showing dose-dependent changes in the mean amplitude ratio in the presence of the blocker to mean amplitude in control. One-way ANOVA followed by Tukey’s multiple comparisons test. Insignificant changes are marked as n/s; *p* < 0.05 (*), *p* < 0.01 (**), *p* < 0.001 (***). (**E**,**E′**) Traces of neurons (**E**) and diagram (**E′**) demonstrating changes in the ratio of mean amplitude in control to mean amplitude in the presence of nifedipine, ML-218, ω-conotoxin MVIIC (blocker of P/Q- and N-type VGCC). N = 100, n = 4. One-way ANOVA followed by Tukey’s multiple comparisons test. *p* < 0.01 (**).

First, we compared the effects of different VGCC blockers on KCl-induced depolarization. Acute elevation of the extracellular K^+^ concentration evokes the depolarization of excitable cells, thus promoting the activation of voltage-gated channels and [Ca^2+^]_i_ increase. It should be noted that depolarization can stimulate the secretion of neurotransmitters. Therefore, to exclude Ca^2+^ inflow through NMDA receptors (NMDARs), AMPA receptors (AMPARs), and kainate receptors (KARs), all experiments were performed in the presence of the antagonists. Figure 1A–C demonstrate that the effects of dihydropyridines significantly differ from the effects of non-dihydropyridines. While verapamil and diltiazem suppress the calcium response of neurons to depolarization in a dose-dependent manner, nifedipine and isradipine suppress the response only by approximately two times. The maximal suppression in the case of verapamil and diltiazem reached a concentration of 300 μM and IC_50_ was 52.5 μM and 51.1 μM, respectively. Further experiments demonstrated that the Ca^2+^ signal observed in neurons in the presence of nifedipine was suppressed by the blockers of T-, N-, and P/Q-type VGCC (ML-218 and ω-conotoxin MVIIC). We used ω-conotoxin MVIIC at a concentration of 1 μM because it blocks N- and P/Q-type VGCC at this dose [29]. In turn, the concentration of ML-218 was 10 μM since ML-218 non-selectively blocks L- and N-type VGCC at higher doses [30].

Thus, several conclusions can be drawn from these experiments. First, the Ca^2+^ response of neurons to depolarization comprises Ca^2+^ inflow through different VGCC, first of all through L-type and T-, N-, and P/Q-type VGCC. Second, the complete suppression of the Ca^2+^ response observed in the presence of verapamil or diltiazem is obviously caused by the non-selective blockade of other VGCC types. This effect possibly occurs at concentrations higher than 10 μM. Third, dihydropyridines (nifedipine and isradipine) selectively block L-type VGCC and do not affect other sources of depolarization-induced Ca^2+^ inflow.

Moreover, as shown in Figure 1, nifedipine and isradipine similarly affect the Ca^2+^ response in neurons. In turn, the effects of diltiazem and verapamil are also similar, but they differ from the effects of the dihydropyridines. Therefore, we chose verapamil and nifedipine to investigate in further experiments the effects of the blockers and the role of L-type VGCC in more detail. The effects of diltiazem and isradipine are shown in the Supplementary Materials (Figure S1).

### 2.2. Contribution of L-Type VGCC to the Epileptiform Activity of Neurons

#### 2.2.1. The Effects of L-Type VGCC Blockade on the Parameters of [Ca^2+^]_i_ Oscillations

**Figure 2:**
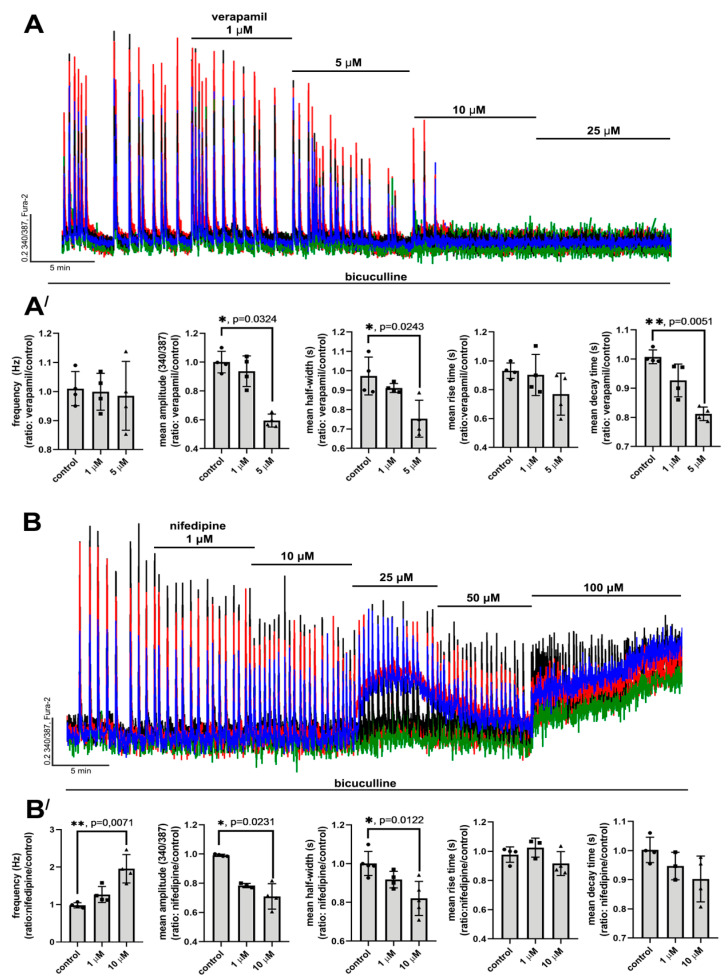
The effects of verapamil and nifedipine on [Ca^2+^]_i_ oscillations. (**A**,**B**) Effects of different doses of verapamil and nifedipine on bicuculline-induced [Ca^2+^]_i_ oscillations. The concentration of bicuculline was 10 μM in all experiments. N = 100, n = 4. (**A′**,**B′**) Scatter plots demonstrating the changes in frequency, amplitude, half-width, and rise and decay times of [Ca^2+^]_i_ oscillations. Data are presented as the ratio of a particular parameter mean value in the presence of the blocker to the value in control. Rise and decay time correspond to time intervals from a basal level of [Ca^2+^]_i_ to peak of the oscillation and vise versa, respectively. Kruskal–Wallis test followed by Dunn’s multiple comparisons test. *p* < 0.05 (*), *p* < 0.01 (**).

**Figure 3:**
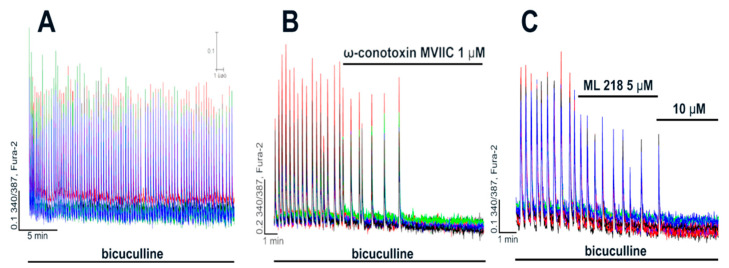
Contribution of T-, N-, and P/Q-type VGCC to generation of [Ca^2+^]_i_ oscillations. (**A**) [Ca^2+^]_i_ oscillations induced by bicuculline (10 μM) in a control experiment. (**B,C**) Effects of ω-conotoxin MVIIC (N- and P/Q-type VGCC blocker) and ML 218 (T-type VGCC blocker) on bicuculline-induced [Ca^2+^]_i_ oscillations. N = 100, n = 4.

Epileptiform activity was induced by GABA(A)R antagonist, bicuculline. Blockade of GABA(A)Rs leads to the disinhibition of neuronal networks, promoting the generation of high-amplitude synchronous [Ca^2+^]_i_ oscillations in all neurons (Figure 2A,B and Figure 3A). These oscillations are caused by the activation of ionotropic glutamate receptors since NMDAR antagonists decrease the amplitude, whereas AMPAR antagonists completely suppress the synchronous [Ca^2+^]_i_ changes in neurons [31,32]. As can be seen in Figure 3A, the frequency and amplitude of [Ca^2+^]_i_ oscillations changed insignificantly in the control experiment during 30 min of bicuculline exposure. Since the duration of all experiments did not exceed 30 min, all changes in [Ca^2+^]_i_ dynamics were induced by the studied blockers.

Figure 2A,A′ show that verapamil (and diltiazem; see Supplementary Figure S2) decreases the amplitude and half-width of [Ca^2+^]_i_ oscillations and suppresses the oscillations at concentrations ≥ 10 μM. Additionally, decay time also decreases in this case. In turn, the frequency of [Ca^2+^]_i_ oscillations and rise time change insignificantly. Nifedipine (and isradipine; see Supplementary Figure S3), similarly to verapamil, decreased the amplitude and half-width of the oscillations (Figure 2B,B′) and did not affect the rise time. However, the difference between the decay time in the control and in the presence of nifedipine was insignificant (Figure 2B′), but the frequency of oscillations substantially increased in this case, which was not observed in the presence of verapamil. In contrast, even high doses of nifedipine do not suppress [Ca^2+^]_i_ oscillations completely. Interestingly, nifedipine at a concentration of 25 μM evokes a significant elevation of basal [Ca^2+^]_i_ in some neurons, while, at a concentration of 100 μM, we observed [Ca^2+^]_i_ elevation in all neurons (Figure 2B).

Thus, these experiments demonstrate that verapamil and nifedipine in the range of concentrations of 1–5 μM and 1–10 μM, respectively, demonstrate similar effects on the [Ca^2+^]_i_ oscillations in neurons, i.e., these drugs decreased the amplitude and half-width of the oscillations. However, the effects of the blockers differ at doses exceeding the indicated ranges. While verapamil suppresses [Ca^2+^]_i_ oscillations, nifedipine, on the contrary, increases the frequency and evokes an irreversible increase in basal [Ca^2+^]_i_ at higher doses.

Complete inhibition of [Ca^2+^]_i_ oscillations in the case of verapamil is evidently explained by the non-specific blockade of other VGCC types at concentrations ≥ 10 μM (Figure 1). Figure 3B shows that ω-conotoxin MVIIC initially decreases the frequency of the oscillations but then completely suppresses them. In turn, ML 218, a blocker of T-type VGCC, also decreases the frequency and completely suppresses [Ca^2+^]_i_ oscillations at higher doses (Figure 3C). Considering the non-specific action of verapamil on N-, T-, and P/Q-type VGCC demonstrated in Figure 1, it may be suggested that the suppression of bicuculline-induced [Ca^2+^]_i_ oscillations at high doses (≥10 μM) is caused by the non-selective blockade. This assumption is confirmed by the fact that even high doses of nifedipine (50–100 μM) do not suppress [Ca^2+^]_i_ oscillations (Figure 2B).

Taking into consideration these data, it can be concluded that the direct effect of L-type VGCC blockade is a decrease in the amplitude and half-width of [Ca^2+^]_i_ oscillations. In this regard, other changes observed at high doses of the blockers are caused by non-selective action. However, it is most likely that even the blockade of all L-type VGCC does not suppress [Ca^2+^]_i_ oscillations.

#### 2.2.2. The Effect of L-Type VGCC Blockade on Paroxysmal Depolarization Shift in Neurons

**Figure 4:**
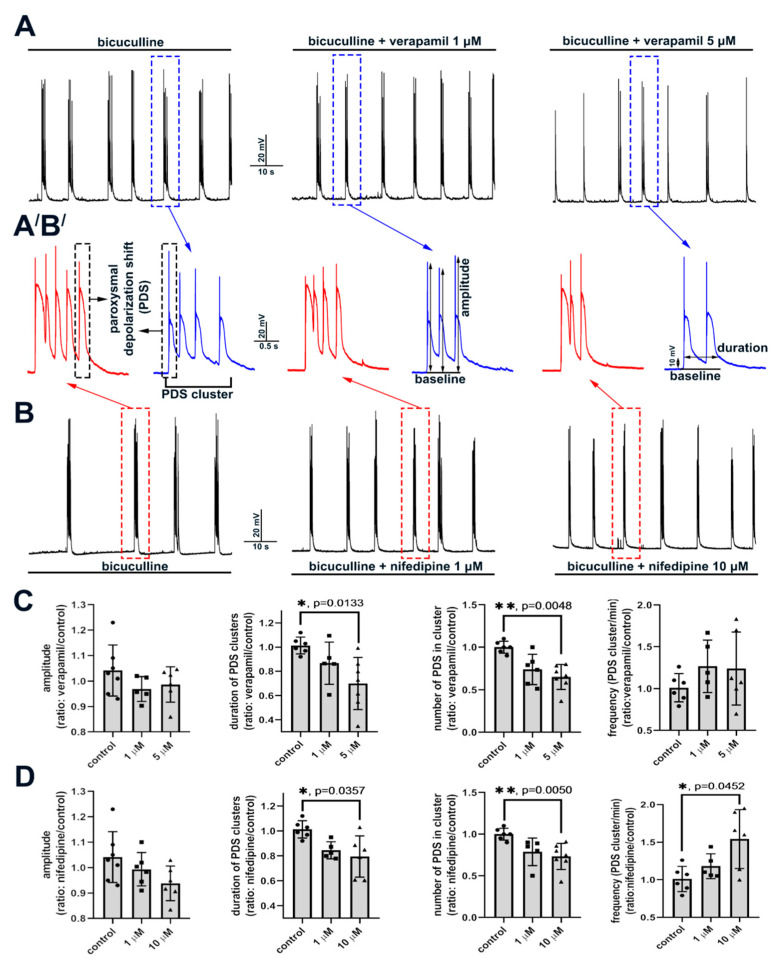
Effects of L-type VGCC blockers on a structure of PDS. (**A**,**B**) Changes in the membrane potential of neurons in the presence of bicuculline (10 μM) and different concentrations of verapamil and nifedipine. (**A′**,**B′**) Enlarged images of individual PDS clusters. (**C**,**D**) Scatter plots demonstrating changes in different PDS parameters. Data are presented as the ratio of a particular parameter mean value in the presence of the blocker to the value in control. Kruskal–Wallis test followed by Dunn’s multiple comparisons test. *p* < 0.05 (*), *p* < 0.01 (**).

High-amplitude bicuculline-induced [Ca^2+^]_i_ oscillations correspond to PDSs registered with a patch-clamp technique (Figure 4). The doses of verapamil and nifedipine selectively blocking L-type VGCC decrease the number of PDSs in a cluster, thus decreasing the cluster duration (Figure 4C,D). The effects of diltiazem are shown in Figure S5 (see Supplementary). However, the amplitude of initial AP in PDSs changes insignificantly. Notably, the number of PDSs in a cluster does not change without any exposure in control experiments (Figure 4A,B,A′B′ left panels). Moreover, it can be concluded that the pattern of PDSs in a cluster does not change in the presence of the blockers. As in the case of [Ca^2+^]_i_ measurements, nifedipine increases the frequency of PDS cluster occurrence (Figure 4D and Figure S4). Thus, blockade of L-type VGCC decreases the number of PDSs in a cluster alongside a decrease in the amplitude and half-width of [Ca^2+^]_i_ oscillations.

### 2.3. Side Effects of High Doses of Dihydropyridines

**Figure 5:**
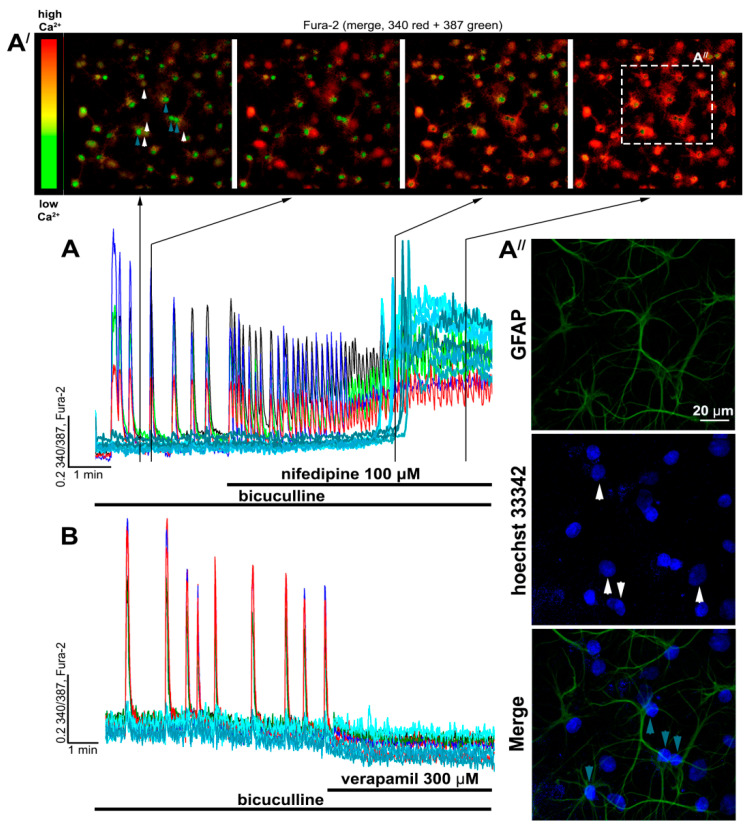
The differences in the effects of high doses of nifedipine and verapamil. (**A**,**B**) The effects of high doses of nifedipine and verapamil on [Ca^2+^]_i_ dynamics in neurons (colored curves) and astrocytes (cyan curves). (**A′**) Changes in [Ca^2+^]_i_ presented as merged 340+387 Fura-2 images. (**A″**) Immunostaining of cells from panel A′ with anti-GFAP antibodies (a marker of astrocytes) and nuclear dye Hoechst 33342. Arrows indicate neurons (white) and astrocytes (blue), with [Ca^2+^]_i_ traces presented in Figure 5A.

As shown in Figure 2B and Figure S3, high doses of nifedipine and isradipine evoke irreversible [Ca^2+^]_i_ elevation in all neurons. In the experiment presented in Figure 5A, a high concentration of nifedipine was added (100 μM). The frequency of [Ca^2+^]_i_ oscillations increased after the blocker application, and then the basal [Ca^2+^]_i_ level was elevated in all neurons. Moreover, we observed [Ca^2+^]_i_ elevation in other cells (not neurons) in the culture (Figure 5A,A′). Immunostaining revealed that cells that responded to nifedipine with basal [Ca^2+^]_i_ elevation without the oscillations were astrocytes since they were stained with antibodies against GFAP (Figure 5A″). In turn, verapamil did not evoke sustained elevations of [Ca^2+^]_i_ in cells, even at a concentration of 300 μM (Figure 5B). Thus, despite the greater selectivity of dihydropyridines towards L-type VGCC, high doses of these drugs affect neurons and astrocytes, leading to an irreversible [Ca^2+^]_i_ increase.

## 3. Discussion

### 3.1. L-Type VGCC Blockers

Here, we compare the effects of four L-type VGCC blockers on the bicuculline-induced epileptiform activity in rat hippocampal cell cultures. We show that verapamil (belonging to the group of phenylalkylamines) and diltiazem (belonging to benzothiazepines) demonstrate similar effects. However, the effects of these two drugs differ from the effects of dihydropyridines, namely nifedipine and isradipine. Verapamil and diltiazem decreased or completely suppressed [Ca^2+^]_i_ oscillations and the KCl-induced response in a dose-dependent manner. In turn, even high doses of dihydropyridines do not suppress these Ca^2+^ events. The fact that Ca_v_1.3 channels are less susceptible to dihydropyridine block than Ca_v_1.2 channels may explain the incomplete suppression observed in our experiments [33,34,35]. However, despite the low susceptibility, dihydropyridines block Ca_v_1.3 channels. High doses of nifedipine (200 μM) and isradipine (10 μM) significantly exceeding IC_50_ for Ca_v_1 channels did not suppress the Ca^2+^ responses. Notably, we did not observe dose-dependent effects of the used dihydropyridines on the response amplitude. An alternative explanation for the incomplete suppression is the assumption that the dihydropyridines block all L-type VGCC, but the remaining (dihydropyridine-insensitive) [Ca^2+^]_i_ increase is mediated by Ca^2+^ inflow through other VGCC types. We confirmed this assumption in experiments with N-, T-, and P/Q-type VGCC blockers. These drugs almost completely suppress dihydropyridine-insensitive Ca^2+^ inflow.

Thus, the dose-dependent suppression of the KCl-induced Ca^2+^ response and bicuculline-induced [Ca^2+^]_i_ oscillations by verapamil and diltiazem is caused by non-selective action on other VGCC types. In this regard, a range of studies demonstrate that diltiazem and verapamil can block other VGCC types [34,36,37,38]. Furthermore, these drugs more efficiently block Ca_v_1.2 than Ca_v_1.3 [34,35].

Although dihydropyridines are more selective towards L-type VGCC, high doses of these blockers induce various side effects. In our experiments, high doses of nifedipine and isradipine induced an irreversible elevation of [Ca^2+^]_i_ in neurons and astrocytes. This phenomenon may be explained by the excessive accumulation or secretion of glutamate in synapses. It was shown that nifedipine enhances Ca^2+^-independent glutamate secretion. Interestingly, blockade of L-type VGCC does not contribute to this effect [39]. The authors indicate that the minimal nifedipine concentration inducing glutamate secretion is 100 nM, while EC_50_ is 7.8 μM.

Thus, it can be concluded from the literature data and our experiments that the studied L-type VGCC blockers selectively block L-type channels only in a particular range of concentrations. Obviously, verapamil and diltiazem block N-, T-, and P/Q-type VGCC at concentrations ≥ 10 μM. In turn, high doses of nifedipine (≥10 μM) and isradipine (≥5 μM) evoke an irreversible [Ca^2+^]_i_ elevation in neurons and astrocytes. L-type VGCC blockers are actively used for the investigation of the properties and functions of L-type channels, and the range of concentrations occasionally exceeds the threshold of non-selective action [33,40,41,42,43,44]. Hence, it is necessary to choose the concentration and interpret the obtained results carefully.

### 3.2. Role of L-Type VGCC in Epileptiform Activity

The present work and our previous study showed that PDSs in neuronal networks are accompanied by high-amplitude [Ca^2+^]_i_ oscillations [32,45]. This paroxysmal activity occurs spontaneously in some cultures or can be induced by different types of exposure, shifting the E/I balance toward excitation. Activation of NMDA and AMPA receptors is required to induce PDSs, thus indicating that PDS generation is a network event resulting from the synchronous excitation of numerous glutamatergic neurons. As we previously showed, regular hyperpolarizing chloride currents occur in neurons in most prepared rat hippocampal cell cultures [45]. In turn, the application of bicuculline suppresses the chloride currents and induces the appearance of inward depolarizing currents and the generation of synchronous [Ca^2+^]_i_ oscillations in all neurons in a network. Hyperpolarizing currents can be caused by GABA release from GABAergic neurons or astrocytes [46]. Thus, although the causes and mechanism of individual PDS generation are well-known. It remains elusive why some PDSs combine into clusters and which factors determine the number of PDSs in a cluster [4,47].

Ca^2+^ inflow through VGCC is considered one of the main contributors to PDS generation and spread [4], whereas the disturbance of Ca^2+^ homeostasis is believed to play a pivotal role in epilepsy development [48,49]. Nevertheless, only a few studies demonstrate the correlation between changes in [Ca^2+^]_i_ in neurons and PDS generation. Furthermore, only a small number of works demonstrate that the amplitude of [Ca^2+^]_i_ oscillations correlates with epileptiform discharges [13,50].

Here, we show that PDS clusters are accompanied by high-amplitude synchronous [Ca^2+^]_i_ oscillations in all neurons. The number of PDSs in a cluster and the frequency of the cluster occurrence change insignificantly without any exposure. This conclusion is also confirmed by the results of [Ca^2+^]_i_ measurements. The obtained data indicate that the number of PDSs in a cluster and the frequency of cluster occurrence are non-stochastic parameters determined by still unrevealed mechanisms. We show that blockade of L-type VGCC decreases the amplitude and half-width of [Ca^2+^]_i_ oscillations and also decreases the number of PDSs in a cluster. However, the frequency of PDS cluster occurrence is not affected by L-type VGCC blockade. An increase in the frequency of [Ca^2+^]_i_ oscillations in the presence of dihydropyridines is obviously caused by non-selective action. Hence, it may be concluded that L-type VGCC plays a pivotal role in maintaining Ca^2+^ inflow during the oscillations, and the duration of the Ca^2+^ pulse possibly determines the number of PDSs in a cluster. It was shown (including in our studies) that the generation of each subsequent PDS may occur both during the depolarizing plateau (high membrane potential) and during the decrease in potential to values close to resting membrane potential [13,32,47]. Thus, it is unlikely that the generation of each subsequent PDS in a cluster is determined by membrane potential. In turn, the level of [Ca^2+^]_i_ increases during each cluster [32,50], whereas, in the case of single APs generating between the clusters, [Ca^2+^]_i_ changes are not observed in the soma of neurons [51]. Notably, PDS is terminated when [Ca^2+^]_i_ decays. Moreover, as with the blockade of L-type VGCC, NMDAR antagonists also decrease the [Ca^2+^]_i_ oscillation amplitude [32]. Therefore, it can be suggested that the level of [Ca^2+^]_i_ determines the number of PDSs in a cluster. Apparently, the source of Ca^2+^ inflow is not important since L-type VGCC or NMDAR inhibition results in similar effects, namely a decrease in the amplitude of [Ca^2+^]_i_ oscillations and the number of PDSs in a cluster. However, the pattern of individual PDSs does not change in either case.

Thus, according to our results and the data of other researchers, the [Ca^2+^]_i_ level determines the number of PDSs in a cluster. However, the correlation between the [Ca^2+^]_i_ level and the generation of PDSs, and the mechanisms determining the number of PDSs in a cluster, remain unclear and require further investigations.

## 4. Materials and Methods

### 4.1. Preparation of Hippocampal Cell Cultures

The detailed protocol of rat hippocampal neuroglial cell culture preparation was described previously [45,52]. Briefly, P0-2 Sprague-Dawley pups were euthanized and decapitated. The extracted hippocampus was placed in a tube with cold Ca^2+^/Mg^2+^-free Versene solution and then carefully minced. Then, the tissue was treated with 1% trypsin solution for 10 min at 37 °C under constant stirring. Then, tissue fragments were gently washed twice with cold Neurobasal-A medium to inactivate trypsin and gently triturated with a pipette. Non-triturated tissue debris was removed, and the suspension was sedimented for 3 min at 2000 rpm. Then, the supernatant was carefully removed, and the pellet was resuspended in a culture medium composed of Neurobasal-A medium supplemented with B-27 (2%) and freshly prepared glutamine (0.5 mM). Penicillin–streptomycin was also added into the culture medium. The suspension was distributed in glass cylinders (100 μL per cylinder) placed on polyethyleneimine-coated glass coverslips. Petri dishes with coverslips were placed in a CO_2_ incubator for 1 h for cell sedimentation and adhesion. After this, the cylinders were removed, and 2 mL of the culture medium was added into the dishes with the coverslips. Cultures were grown in a CO_2_ incubator at 37 °C and 95% humidity for two weeks and then were used in experiments. We used 12–14 DIV (days *in vitro*) cultures in all experiments.

### 4.2. Fluorescent [Ca^2+^]_i_ Measurements

The changes in intracellular Ca^2+^ concentration ([Ca^2+^]_i_) were evaluated using ratiometric calcium probe Fura-2 AM. The cells were stained for 40 min at 28 °C with the probe (working concentration 3 μM) dissolved in Hank’s balanced salt solution composed of (in mM): 156 NaCl, 3 KCl, 0.8 MgSO_4_, 1.25 KH_2_PO_4_, 0.35 Na_2_HPO_4_, 1.5 CaCl_2_, 10 glucose, and 10 HEPES, pH 7.35. For ratiometric [Ca^2+^]_i_ measurements, we used a Leica DMI 6000B fluorescent microscope (Leica Microsystems, Wetzlar, Germany) equipped with a Fura-2 external filter wheel (BP340/30 and BP387/15 filters) for fast ratiometric acquisition and internal FURA-2 filter cube (dichroic mirror 72100bs, emission filter HQ 540/50m). Time-lapse images were obtained with a CCD camera, Hamamatsu C9100 (Hamamatsu Photonics K.K., Hamamatsu City, Japan). We used ImageJ (NIH, Bethesda, MD, USA) software for image data processing, following the previously reported data analysis protocol [45]. Changes in [Ca^2+^]_i_ are expressed as 340/387 ratio.

### 4.3. Immunocytochemistry

Immunostaining was performed as described previously [45,52,53]. Briefly, after [Ca^2+^]_i_ measurements, the cultures were washed three times with PBS and fixed with freshly prepared 4% paraformaldehyde solution for 20 min. After this, the cells were triply rinsed with ice-cold PBS and incubated for 30 min at room temperature with 10% goat serum + 0.1% Triton X-100 in PBS to block non-specific binding of antibodies. After this, cells were stained overnight at 4 °C with mouse anti-GFAP antibodies diluted 1:200 in PBS containing 1% of goat serum and 0.1% trypsin. Primary antibodies were washed three times (each wash was 5 min) with PBS, and then the cells were incubated with secondary goat anti-mouse antibodies conjugated with Alexa Fluor 647. After this, the cultures were washed three times with PBS, and the secondary antibodies’ fluorescence was detected with a Leica TCS SP5 confocal microscope (Leica Microsystems, Wetzlar, Germany). To probe the nuclei of cells, we used Hoechst 33,342 (5 μg/mL). The developed technique of matching the images of vital [Ca^2+^]_i_ measurements and postvital immunostaining was described in detail in our previous works [45,52].

### 4.4. Whole-Cell Patch-Clamp

Changes in membrane potential in neurons were registered with the whole-cell patch-clamp technique. All experiments were performed at 28 °C in HBSS solution. The internal solution contained (in mM): 10 KCl, 125 K-gluconate, 1 MgCl_2_ × 6H_2_O, 0.25 EGTA, 10 HEPES, 2 Na_2_-ATP, 0.3 Mg-ATP, 0.3 Na-GTP, 10 Na_2_-phosphocreatine (pH 7.2). Data were recorded with an Axopatch 200B amplifier and low-noise data acquisition system, the Axon DigiData 1440A digitizer (Molecular Devices, San Jose, CA, USA), using pCLAMP 10 software (Molecular Devices, San Jose, CA, USA).

### 4.5. Statistics and Data Analysis

OriginLab Pro 2016 (OriginLab, Northampton, MA, USA), MS Excel (Microsoft Corporation, Redmond, WA, USA), and Prism GraphPad 8 (GraphPad Software, San Diego, CA, USA) software were used for data and statistical analysis. The parameters of [Ca^2+^]_i_ oscillations (amplitude, half-width, rise time, decay time) were calculated with ClampFit 10 (Molecular Devices, San Jose, CA, USA). Thresholds (Min and Max) for rise and decay time calculations were 10 and 90%. We used one-way ANOVA followed by Tukey’s multiple comparisons test and Kruskal–Wallis test followed by Dunn’s multiple comparisons test for group comparisons. N—the number of analyzed cells in an experiment; n—the number of repeats.

### 4.6. Reagents

The reagents that were used in experiments are listed below: Paraformaldehyde (P6148), Poly(ethyleneimine) solution (P3143), penicillin–streptomycin (P4333), (+)-cis-Diltiazem hydrochloride (D2521), (±)-Verapamil hydrochloride (V4629), Isradipine (I6658), Nifedipine (N7634) (Sigma-Aldrich, Saint Louis, MO, USA), Neurobasal-A medium (10888022), B-27 supplement (17504044), Trypsin 2.5% (15090046), Goat serum New Zeland origin (16210072) (Life Technologies, Grand Island, NY, USA), Fura-2 AM (F1221), Hoechst 33,342 Trihydrochloride Trihydrate (H1399) (Molecular Probes, Eugene, OR, USA), ML 218 hydrochloride (4507) (Tocris Bioscience, Bristol, UK), NBQX disodium salt (N-186), D-AP5 (D-145) (Alomone Labs, Jerusalem, Israel), Bicuculline (11727) (Cayman Chemical, Ann Arbor, MI, USA), goat anti-mouse Alexa Fluor 647 antibody (ab150115) (Abcam, Cambridge, UK), monoclonal mouse anti-GFAP antibodies (GF1 clone, L18/03) (Bialexa, Moscow, Russian Federation); Triton X-100 (Am-O694) (Amresco LLC, Solon, OH, USA); EGTA (A-0878), EDTA (A5097), (AppliChem, Darmstadt, Germany); HEPES (Cat. No 3350) (Dia-M, Moscow, Russian Federation).

## Data Availability

The data presented in this study are available on request from the corresponding author.

## Supplementary Materials

The following are available online at https://www.mdpi.com/article/10.3390/ijms221910342/s1.
