# Supplementary material for: Role of L-Type Voltage-Gated Calcium Channels in Epileptiform Activity of Neurons
Source: Int J Mol Sci. 2021 Sep 25;22(19):10342. doi: 10.3390/ijms221910342 (PMC8508770; doi:10.3390/ijms221910342)
Supplement: Supplementary file 1 [file ijms-22-10342-s001.zip › ijms-1375967-supplementary.pdf]

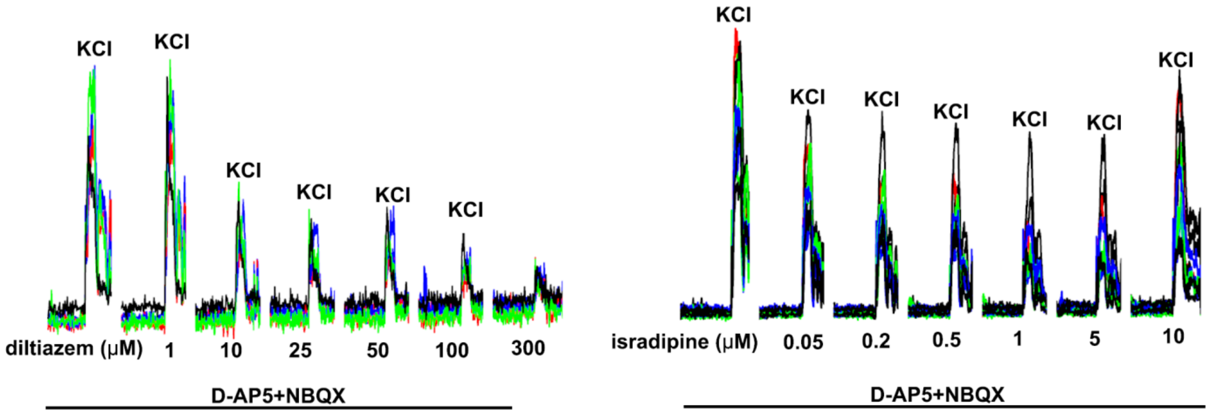

Figure S1. The response of neurons to depolarization induced by 35 mM KCl in the presence of different doses of diltiazem and isradipine. The experiments were performed in the presence of AMPAR/KAR and NMDAR antagonists, NBQX (10 μM) and D-AP5 (10 μM), respectively.

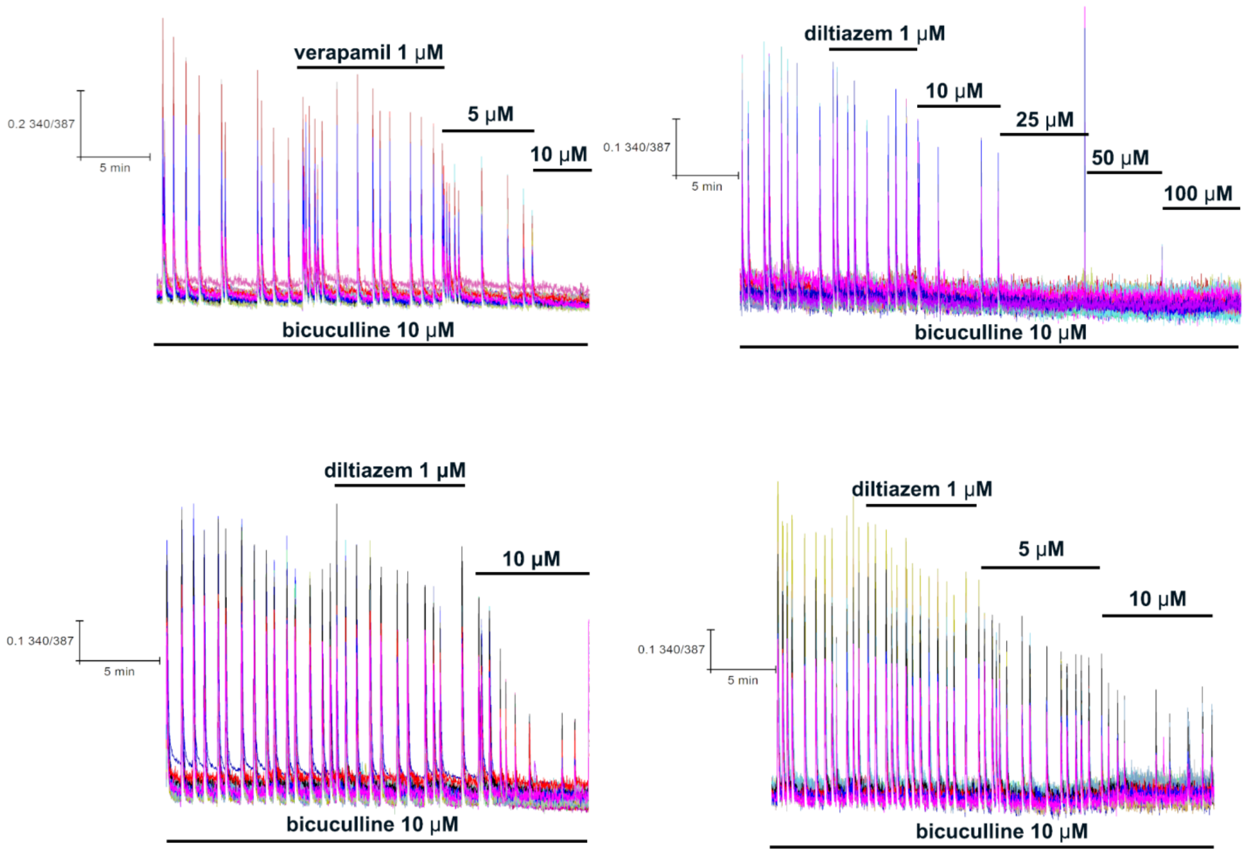

Figure S2. The effects of different concentrations of verapamil and diltiazem on bicuculline-induced (10 μM) $[Ca^{2+}]_i$ oscillations in neurons.

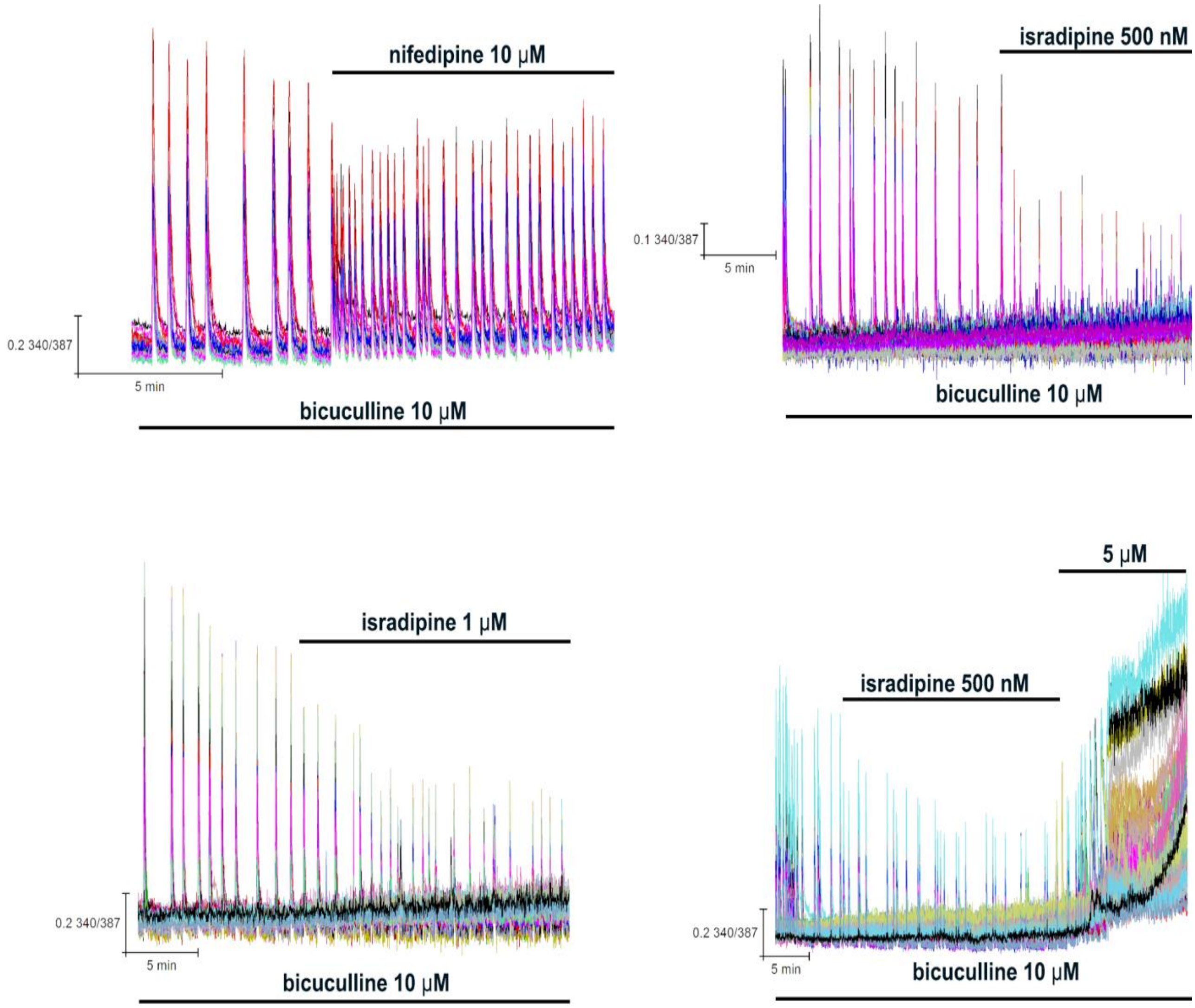

Figure S3. The effects of different concentrations of nifedipine and isradipine on bicuculline-induced (10 μM) $[Ca^{2+}]_i$ oscillations in neurons.

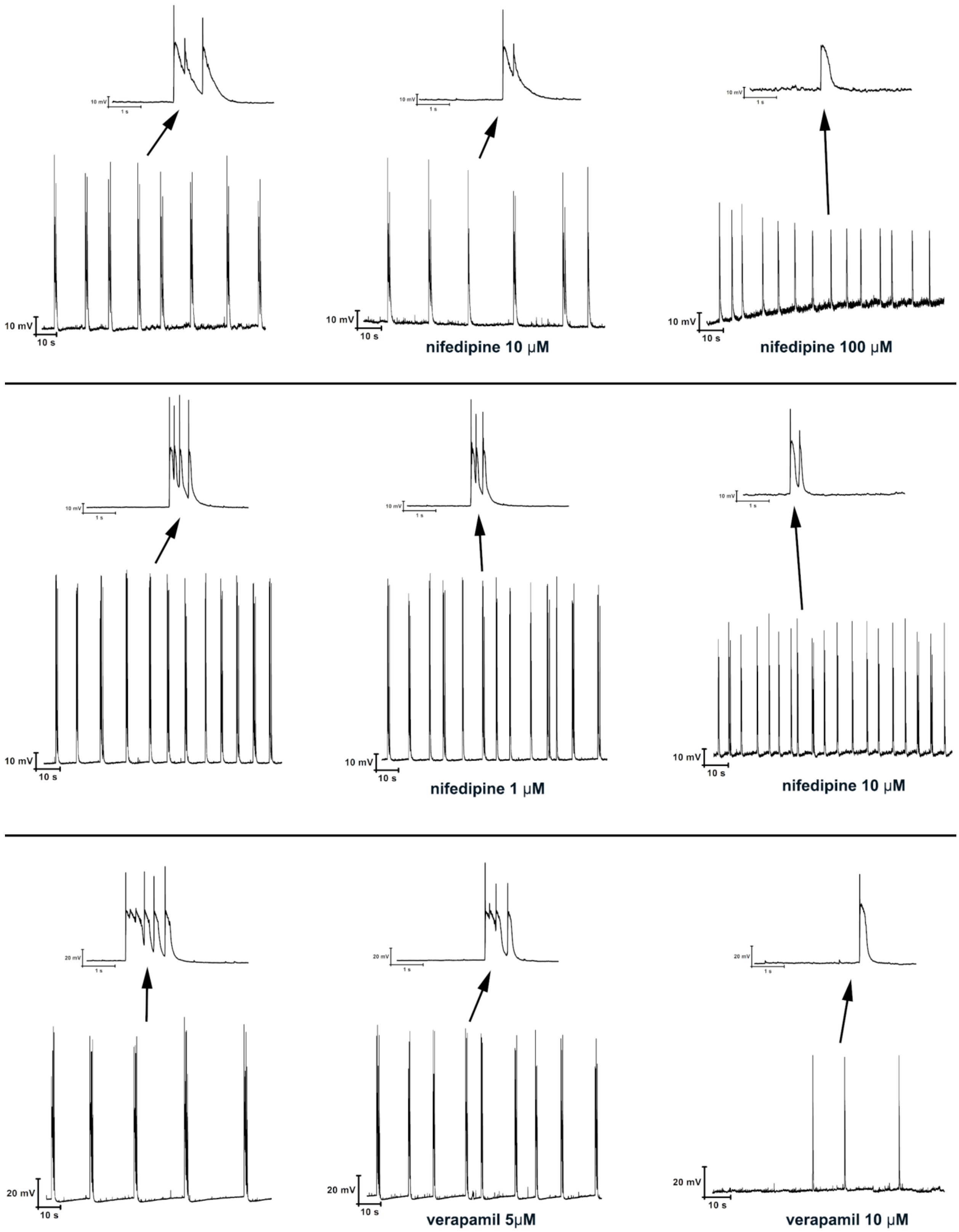

Figure S4. The effects of different doses of nifedipine and verapamil on bicuculline-induced PDSs. Black arrows indicate individual PDS clusters. All recordings were performed in whole-cell configuration.

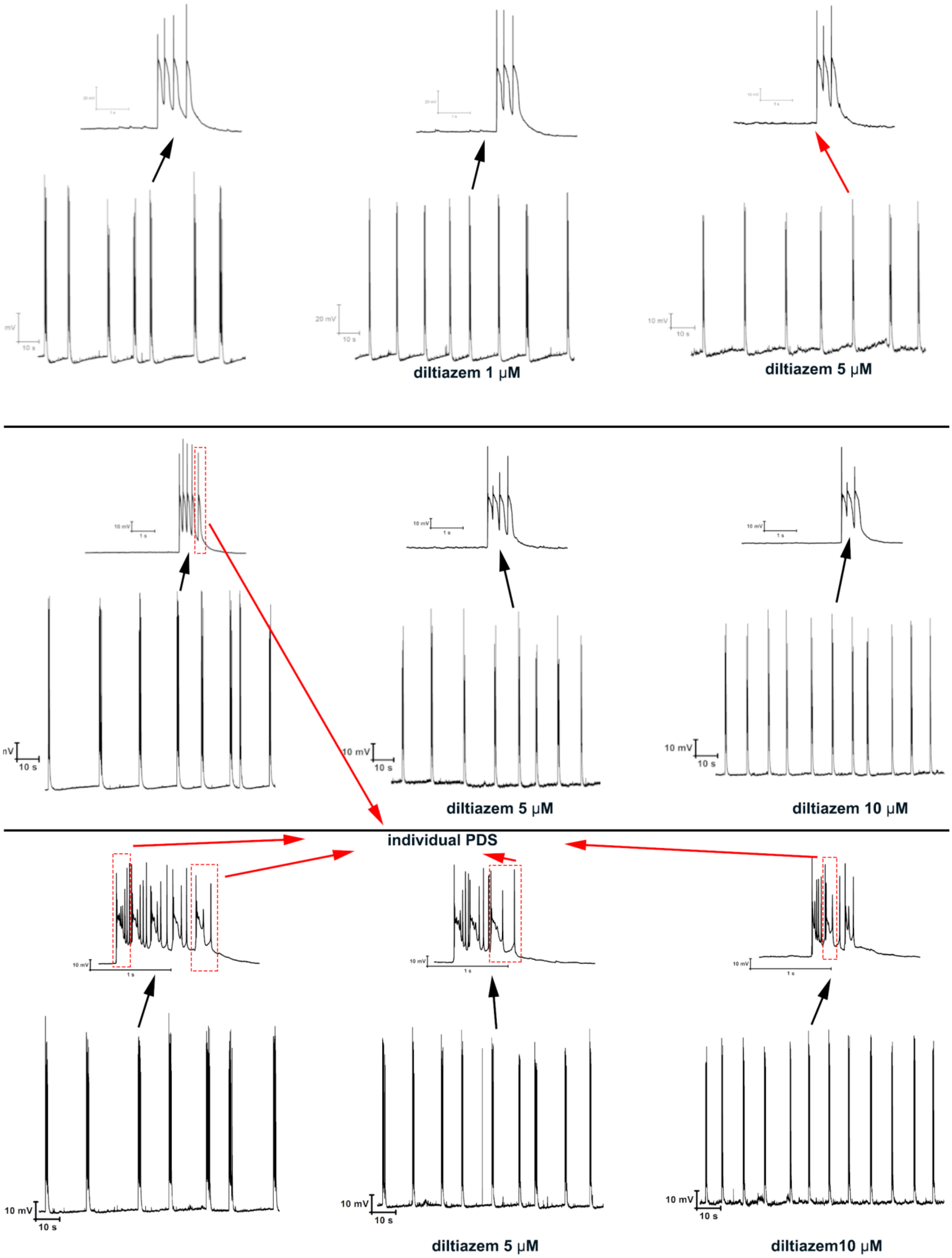

Figure S5. The effects of different doses of diltiazem on bicuculline-induced PDSs. Black arrows indicate individual PDS clusters. All recordings were performed in whole-cell configuration.
